# Prognostic models for estimating severity of disease and predicting 30-day mortality of *Hypervirulent Klebsiella pneumoniae* infections: a bicentric retrospective study

**DOI:** 10.1186/s12879-023-08528-x

**Published:** 2023-08-25

**Authors:** Jieen Huang, Yanzhu Chen, Ming Li, Shujin Xie, Huasheng Tong, Zhusheng Guo, Yi Chen

**Affiliations:** 1 Department of Intensive Care Medicine, Binhaiwan Central Hospital of Dongguan, No.111, Humen Road, Humen Town, Dongguan City, Guangdong Province China; 2 Department of Respiratory and Critical Care Medicine, The First Affiliated Hospital, Sun Yat-sen University, Guangzhou, Guangdong Province China; 3 Department of Laboratory Medicine, Binhaiwan Central Hospital of Dongguan, Dongguan City, Guangdong Province China; 4 Department of Laboratory Medicine, Dongguan Tungwah Hospital, No.1, Dongcheng East Road, Dongguan City, Guangdong Province China; 5 Department of Emergency Medicine, General Hospital of Southern Theatre Command, No. 919, Renmin North Road, Yuexiu District, Guangzhou City, Guangdong Province China

**Keywords:** *Hypervirulent Klebsiella pneumoniae*, Infections, Prognostic model, Severity, Mortality

## Abstract

**Background:**

*Hypervirulent Klebsiella pneumoniae* (*hvKP*) is emerging globally and can cause various, severe infections in healthy individuals. However, the clinical manifestations of *hvKP* infections are nonspecific, and there is no gold standard for differentiating *hvKP* strains. Our objective was to develop prognostic models for estimating severity of disease and predicting 30-day all-cause mortality in patients with *hvKP* infections.

**Methods:**

We enrolled 116 patients diagnosed with *hvKP* infections and obtained their demographic and clinical data. Taking septic shock and acute respiratory distress syndrome (ARDS) as the primary outcomes for disease severity and 30-day all-cause mortality as the primary outcome for clinical prognosis, we explored the influencing factors and constructed prognostic models.

**Results:**

The results showed that increased Acute Physiologic and Chronic Health Evaluation (APACHE) II score [odds ratio (OR) = 1.146; 95% confidence interval (CI), 1.059–1.240], decreased albumin (ALB) level (OR = 0.867; 95% CI, 0.758–0.990), diabetes (OR = 9.591; 95% CI, 1.766–52.075) and high procalcitonin (PCT) level (OR = 1.051; 95%CI, 1.005–1.099) were independent risk factors for septic shock. And increased APACHE II score (OR = 1.254; 95% CI, 1.110–1.147), community-acquired pneumonia (CAP) (OR = 11.880; 95% CI, 2.524–55.923), and extrahepatic lesion involved (OR = 14.718; 95% CI, 1.005–215.502) were independent risk factors for ARDS. Prognostic models were constructed for disease severity with these independent risk factors, and the models were significantly correlated with continuous renal replacement therapy (CRRT) duration, vasopressor duration, mechanical ventilator duration and length of ICU stay. The 30-day all-cause mortality rate in our study was 28.4%. Younger age [hazard ratio (HR) = 0.947; 95% CI, 0.923–0.973)], increased APACHE II score (HR = 1.157; 95% CI, 1.110–1.207), and decreased ALB level (HR = 0.924; 95% CI, 0.869–0.983) were the independent risk factors for 30-day all-cause mortality. A prediction model for 30-day mortality was constructed, which had a good validation effect.

**Conclusions:**

We developed validated models containing routine clinical parameters for estimating disease severity and predicting 30-day mortality in patients with *hvKP* infections and confirmed their calibration. The models may assist clinicians in assessing disease severity and estimating the 30-day mortality early.

## Introduction

*Hypervirulent Klebsiella pneumoniae* (*hvKP*) has become a global pathogen in recent years [1]. *hvKP*, an invasive type of *Klebsiella pneumoniae* (*KP*), is characterized by severely invasive community-acquired infections in young and immunocompetent individuals with rare sites of infections, rapid progression, severe disease and poor prognosis [1–5].

Currently, the *hvKP* strain is differentiated from *classic Klebsiella pneumoniae* (*cKP*) based on some phenotypic, genotypic properties and determining factors [6]. Li G et al. reported that *Galleria mellonella* killing assay in conjugation with the string test could be used to accurately assess *KP* virulence and differentiate *hvKP* from *cKP* strains [7]. Russo TA et al. noted that *peg-344*, *iroB, iucA*, *prmpA*, *prmpA2*, and siderophore production greater than 30 μg/ml could accurately identify *hvKP* strains [1, 8, 9]. However, there is no universal standard for identifying all *hvKP* strains [10]. Furthermore, detections of genotype and the determining factors are not widely available, especially in developing countries, making it difficult to recognize *hvKP* infections early.

Clinical manifestations of *hvKP* infections, lacking specificity, vary upon the organ involved. Clinically, some patients with *hvKP* infections soon develop to to septic shock, acute respiratory distress syndrome (ARDS), multiorgan failure and death at final. Early identification of *hvKP* infections and prediction of disease severity and outcomes are crucial to improve the survival of *hvKP-*infected patients. Previous studies showed that risk factors for mortality included gastrointestinal fistula, increased Acute Physiology and Chronic Health Evaluation (APACHE) II score and Pitt bacteraemia score, metastatic infection, septic shock, acute respiratory failure and gas formation on imaging [10–12]. To date, there is few report on risk factors for disease severity. Most of the existing studies of *hvKP* mainly focus on virulence factors or drug resistance factors at the genetic level, and little attention has been paid to clinical aspects, especially the disease assessment and prognosis models. Therefore, we concentrated on clinical aspects, retrospectively analyzed the demographic and clinical data of *hvKP-*infected patients to determine the risk factors and tried to construct the prognostic models for disease severity and prognosis.

## Materials and methods

### Study setting and design

Patients with *hvKP* infections firstly diagnosed at Binhaiwan Central Hospital of Dongguan and Dongguan Tungwah Hospital from September 2017 to September 2022, meeting the inclusion and exclusion criteria, were enrolled in this retrospective study. Demographic and clinical data were collected by two individuals. The protocol for this study was approved by the Medical Ethics Committee of Binhaiwan Central Hospital of Dongguan (No. 2021014).

### Inclusion and exclusion criteria

The inclusion criteria were as follows:1. *KP-*infected patients with string test positive. 2. *KP* strains with one or more of genotype (*rmpA*, *rmpA2*, *iucA*, *iroB*, *magA* and *peg344*) positive [8, 13, 14]*.* 3. patients with complete clinical data.

The exclusion criteria were as follows: 1. Patients younger than 18 years old. 2. Patients giving up an active rescue. 3. Immunocompromised patients with history of malignancy (under treatment or in remission for less than five years), immunosuppressive disorders (congenital/acquired immunocompromise), use of immunosuppressive regimens (corticosteroid therapy 1 mg/kg/day prednisone equivalent or corticosteroid therapy for longer than one month, use of another immunosuppressant drug in a high dosage or for longer than one month) [15–17].

### Detection of virulence-associated features and genes

Hypermucoviscosity was identified by the positive string test. A positive string test was defined as the formation of a viscous string > 5 mm in length when bacterial colonies on an agar plate were stretched with an inoculation loop [3]. All *KP* isolates were stored at − 80 °C until they were sent to relevant institutions (Guangzhou Huayin Health Medical Group Co., Ltd.) for detection of virulence-associated features through targeted next-generation sequencing. The genotypic analysis was investigated by polymerase chain reaction with previously described primers [13]. High-throughput sequencing was performed using the Illumina MiSeq Reagent Nano Kit. The reads that were correctly aligned at both ends were compared with the reference gene sequence of each virulence gene in the virulence gene data, and finally, the number of reads for each virulence gene in each sample was obtained.

### Variables collection and definition

Clinical features including age, gender, history of smoking or alcohol consumption, community acquired pneumonia (CAP), comorbidities [diabetes mellitus, hepatopathy, chronic kidney disease (CKD), cardiovascular disease], septic shock, ARDS, and APACHE II score were collected. Laboratory data within 24 h after admission were as follows: white blood cell count (WBC), neutrophil count (NEUT#), lymphocyte count (LYMPH#), monocyte count (MONO#), hemoglobin (HGB), platelet (PLT), C-reactive protein (CRP), procalcitonin (PCT), alanine aminotransferase (ALT), aspartate aminotransferase (AST), total bilirubin (TBIL), direct bilirubin (DBIL), albumin (ALB), glucose (GLU), creatinine (CREA), coagulation plasma prothrombin time (PT), activated partial thromboplastin time (APTT), fibrinogen (FIB), partial pressure of oxygen (PO2), partial pressure of carbon dioxide (PCO2), fraction of inspired oxygen (FIO2), oxygenation index (OI), positive end expiratory pressure (PEEP) and lactate (LAC). Data on lesions and antimicrobial regimens were as follows: infection lesion, number of lesions, number of pathogens, hepatic abscess, pulmonary abscess, bacteremia, initial antimicrobial regimens [piperacillin/third generation of cephalosporins (ceftazidime, ceftriaxone, cefixime), piperacillin/third generation of cephalosporins combined with beta-lactamase inhibitor (cefoperazone-sulbactam, piperacillin-tazobactam, cefotaxime-sulbactam), carbapenemes (meropenem, imipenem, biapenem), quinolones (levofloxacin, moxifloxacin), aminodycosides (amikacin), second generation of cephalosporins (cefamandole, cefuroxime)], and number of antimicrobials. Clinical outcomes: continuous renal replacement therapy (CRRT) duration, vasopressors duration, mechanical ventilator duration, length of intensive care unit (ICU) stay, length of hospital stay and 30-day survival status. Indices of CRRT, vasopressors, mechanical ventilator and ICU were defined as CRRT duration/length of hospital stay, vasopressors duration/length of hospital stay, mechanical ventilator duration/length of hospital stay, and length of ICU stay/length of hospital stay, respectively.

The primary outcomes for the severity of disease were septic shock and ARDS, while the primary outcome for the clinical prognosis was 30-day all-cause mortality.

### Statistical analysis

SPSS software (version 13.0) was used for data analysis. Normally and nonnormally distributed continuous variables were summarized as the mean ± standard deviation (SD) and the median with interquartile range (IQR), respectively. Continuous variables were compared using Student’s t test or the Mann–Whitney U test, and categorical variables were analyzed by using the χ2 test or Fisher’s exact test. *P* < 0.05 was considered statistically significant.

Univariate and multivariate logistic and Cox regression analyses were used to evaluate the risk factors. Variables with *P* < 0.05 in the univariate analysis were analyzed in the multivariate model using the likelihood-ratio test. R software (version 4.2.1, CRAN) was used for the nomogram, validation calibration curve, forest plot, scatterplot, receiver operating characteristic (ROC) curve and Kaplan–Meier (K-M) curve.

## Results

### Clinical features of the patients

**Fig. 1:**
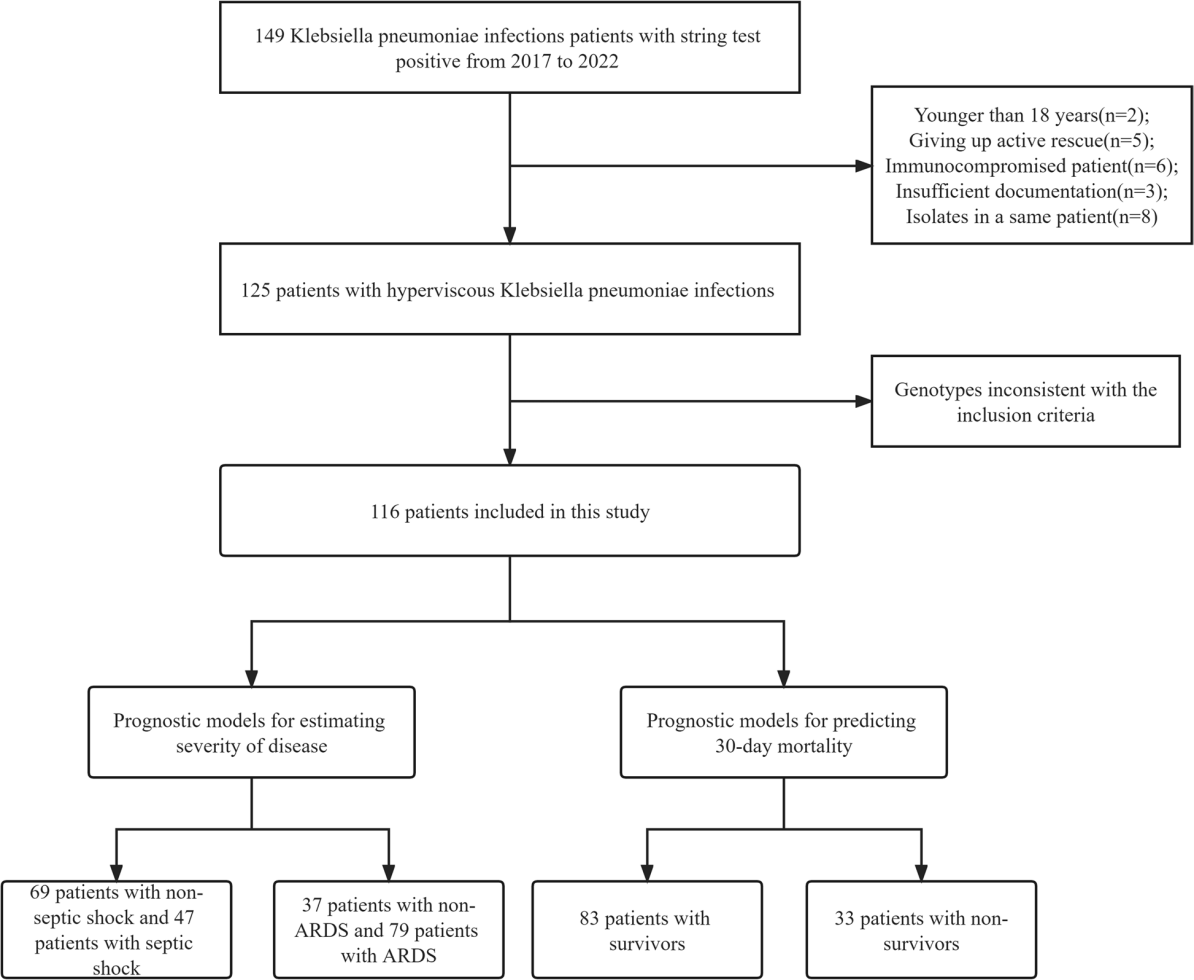
Flowchart of excluded and included patients. Abbreviations: ARDS, acute respiratory distress syndrome

A total of 116 patients were enrolled in our study (Fig. 1), their average age was 55.94 ± 15.93 years and 83 (71.6%) patients were male (Table 1). No significant differences were observed in age, gender, history of smoking/alcohol consumption, CAP, or comorbidities (diabetes mellitus, hepatopathy, CKD, cardiovascular disease) between the two hospitals.

**Table 1:** Clinical variables associated with septic shock and ARDS of *hvKP* infections

Characteristics	Total *n* = 116	Non-septic shock *n* = 69(59.5%)	septic shock *n* = 47(40.5%)	*P* value of septic shock	Non-ARDS *n* = 37(31.9%)	ARDS *n* = 79(68.1%)	*P* value of ARDS
	**(Mean ± SD) or Median (IQR) or n (%)**				**(Mean ± SD) or Median (IQR) or n (%)**		
Age (y)	55.94 ± 15.93	55.09 ± 15.49	57.19 ± 16.64	0.487	59.70 ± 16.12	54.18 ± 15.63	0.082
Gender				0.495			0.125
Female	33(28.4)	18(26.1)	15(31.9)		14(37.8)	19(24.1)	
Male	83(71.6)	51(73.9)	32(68.1)		23(62.2)	60(75.9)	
Smoking				0.635			0.218
No	89(76.7)	54(78.3)	35(74.5)		31(83.8)	58(73.4)	
Yes	27(23.3)	15(21.7)	12(25.5)		6(16.2)	21(26.6)	
Alcohol consumption				0.342			0.210
No	96(82.8)	59(85.5)	37(78.7)		33(89.2)	63(79.7)	
Yes	20(17.2)	10(14.5)	10(21.3)		4(10.8)	16(20.3)	
CAP				0.003			0.000
No	35(30.2)	28(40.6)	7(14.9)		23(62.2)	12(15.2)	
Yes	81(69.8)	41(59.4)	40(85.1)		14(37.8)	67(84.8)	
Diabetes				0.000			0.089
No	75(64.7)	56(81.2)	19(40.4)		28(75.7)	47(59.5)	
Yes	41(35.3)	13(18.8)	28(59.6)		9(24.3)	32(40.5)	
Hepatopathy				0.682			0.441
No	79(68.1)	48(69.6)	31(66.0)		27(73.0)	52(65.8)	
Yes	37(31.9)	21(30.4)	16(34.0)		10(27.0)	27(34.2)	
CKD				1.000			0.968
No	108(93.1)	64(92.8)	44(93.6)		35(94.6)	73(92.4)	
Yes	8(6.9)	5(7.2)	3(6.4)		2(5.4)	6(7.6)	
Cardiovascular disease				0.278			0.037
No	62(53.4)	34(49.3)	28(59.6)		25(67.6)	37(46.8)	
Yes	54(46.6)	35(50.7)	19(40.4)		12(32.4)	42(53.2)	
APACHE II score	20.04 ± 12.51	14.87 ± 8.83	27.64 ± 13.30	0.000	9.46 ± 5.18	25.00 ± 11.84	0.000
WBC (10^9^/L)	14.88(9.58,20.98)	14.83(11.26,17.63)	15.98(7.33,24.74)	0.752	14.93(10.50,16.63)	14.70(9.29,22.14)	0.613
NEUT# (10^9^/L)	12.45(7.87,17.22)	12.25(8.95,14.52)	13.49(6.20,21.16)	0.402	12.51(8.85,14.23)	12.43(6.28,19.63)	0.768
LYMPH# (10^9^/L)	1.03(0.55,1.77)	1.15(0.68,1.82)	0.92(0.42,1.76)	0.049	1.12(0.82,1.67)	1.01(0.46,1.81)	0.371
MONO# (10^9^/L)	0.67(0.29,0.99)	0.76(0.42,1.13)	0.37(0.15,0.94)	0.019	0.76(0.67,1.18)	0.45(0.25,0.92)	0.045
HGB (g/L)	127.50(112.00,141.00)	131.00(115.00,142.00)	122.00(101.00,137.00)	0.065	122.00(114.00,136.00)	128.00(111.00,144.00)	0.340
PLT (10^9^/L)	207.50(122.00,303.25)	216.00(175.00,306.00)	155.00(66.00,264.00)	0.005	201.00(158.00,311.00)	211.00(101.00,300.50)	0.414
CRP (mg/L)	101.62(6.14,184.64)	18.45(1.99,141.09)	141.68(54.23,200.00)	0.001	116.59(24.16,200.00)	64.70(5.00,181.15)	0.434
PCT (ng/mL)	3.33(0.22,30.83)	0.35(0.07,6.60)	19.07(2.95,42.01)	0.000	2.99(0.25,32.57)	3.33(0.21,31.70)	0.828
ALT (U/L)	44.75(19.38,75.08)	37.95(18.45,81.25)	47.90(21.25,74.00)	0.482	49.00(23.00,84.00)	42.00(19.00,74.00)	0.393
AST (U/L)	38.0(23.5,89.75)	38.00(22.00,75.00)	46.00(28.95,102.50)	0.098	40.00(26.00,75.00)	38.00(22.85,98.50)	0.879
TBIL (μmol/L)	13.40(9.48,21.98)	11.11(9.00,18.20)	16.00(10.70,27.40)	0.022	14.30(9.50,24.80)	12.10(9.40,19.80)	0.460
DBIL (μmol/L)	5.89(3.70,10.64)	5.03(2.91,6.89)	8.00(5.09,11.90)	0.001	6.40(4.00,12.70)	5.86(3.35,10.00)	0.339
ALB (g/L)	34.35 ± 6.86	37.32 ± 6.13	30.09 ± 5.50	0.000	37.18 ± 5.40	33.05 ± 7.10	0.004
GLU (mmol/L)	9.42(6.92,15.90)	8.47(6.80,10.50)	12.54(7.17,18.61)	0.006	7.13(6.23,9.72)	10.81(7.64,17.59)	0.001
CREA (μmoI/L)	86.50(66.45,135.64)	74.10(63.35,101.25)	110.100(84.40,176.20)	0.004	82.25(65.62,98.63)	90.10(66.70,157.11)	0.185
PT (s)	12.40(11.40,14.10)	11.65(10.90,12.68)	13.70(11.90,15.70)	0.000	11.70(10.80,13.00)	12.55(11.53,15.03)	0.006
APTT (s)	30.70(24.30,42.30)	27.10(22.65,31.87)	37.40(29.40,47.00)	0.000	27.80(22.50,32.00)	32.45(25.83,45.80)	0.006
FIB (g/L)	4.13(2.80,6.13)	3.72(2.42,6.23)	5.00(3.41,6.13)	0.235	5.00(3.45,6.44)	4.06(2.54,6.01)	0.151
Abscess				0.180			0.002
No	82(70.7)	52(75.4)	30(63.8)		19(51.4)	63(79.7)	
Yes	34(29.3)	17(24.6)	17(36.2)		18(48.6)	16(20.3)	
Hepatic abscess				0.635			0.003
No	89(76.7)	54(78.3)	35(74.5)		22(59.5)	67(84.8)	
Yes	27(23.3)	15(21.7)	12(25.5)		15(40.5)	12(15.2)	
Pulmonary abscess				0.126			0.550
No	113(97.4)	69(100.0)	44(93.6)		37(100.0)	76(96.2)	
Yes	3(2.6)	0(0.0)	3(6.4)		0(0.0)	3(3.8)	
Bacteremia				0.000			0.248
No	76(65.5)	57(82.6)	19(40.4)		27(73.0)	49(62.0)	
Yes	40(34.5)	12(17.4)	28(59.6)		10(27.0)	30(38.0)	
Infection lesion 1				1.000			0.007
Localized intrahepatic lesion	11(9.5)	7(10.1)	4(8.5)		8(21.6)	3(3.8)	
Extrahepatic lesion involved	105(90.5)	62(89.9)	43(91.5)		29(78.4)	76(96.2)	
Infection lesion 2				0.001			0.112
Localized intrapulmonary lesion	50(43.1)	39(56.5)	11(23.4)		12(32.4)	38(48.1)	
Extrapulmonary lesion involved	66(56.9)	30(43.5)	36(76.6)		25(67.6)	41(51.9)	
Number of lesions				0.000			0.098
Single lesion	72(62.1)	54(78.3)	18(38.3)		27(73.0)	45(57.0)	
Multiple lesions	44(37.9)	15(21.7)	29(61.7)		10(27.0)	34(43.0)	
Number of pathogens				0.454			0.002
Single pathogen	81(69.8)	50(72.5)	31(66.0)		33(89.2)	48(60.8)	
Multiple pathogens	35(30.2)	19(27.5)	16(34.0)		4(10.8)	31(39.2)	
CRRT duration(d)	0.00(0.00,1.28)	0.00(0.00,0.00)	0.75(0.00,4.92)	0.000	0.00(0.00,0.00)	0.00(0.00,3.13)	0.000
Vasopressors duration(d)	0.00(0.00,4.24)	0.00(0.00,0.00)	5.00(1.74,10.00)	0.000	0.00(0.00,0.00)	1.79(0.00,7.92)	0.000
Mechanical ventilator duration(d)	2.07(0.00,8.79)	0.75(0.00,6.17)	4.71(1.38,10.96)	0.002	0.00(0.00,0.00)	5.17(1.75,10.96)	0.000
Length of ICU stay(d)	6.50(0.25,15.00)	3.00(0.00,11.50)	7.00(4.00,17.00)	0.002	0.00(0.00,1.50)	9.00(4.00,18.00)	0.000
Length of hospital stay(d)	18.00(9.00,33.00)	20.00(11.00,32.00)	17.00(5.00,33.00)	0.115	17.00(10.50,23.50)	23.00(7.00,37.00)	0.198

### Risk factors associated with disease severity

#### Risk factors associated with septic shock

47/116 (40.5%) patients developed septic shock. The median diagnosis time was 11.63 (4.00, 26.00) hours after admission. There were no significant differences in smoking, alcohol consumption, hepatopathy, CKD or cardiovascular disease between the non-septic shock and septic shock cohorts (*P* > 0.05). Compared with patients with non-septic shock, the septic shock patients had higher levels of APACHE II score, CRP, PCT, TBIL, DBIL, GLU, CREA, PT and APTT, but lower levels of LYMPH#, MONO#, PLT and ALB. Additionally, there were significantly higher proportions of septic shock patients with CAP, diabetes, bacteremia, extrapulmonary lesion involved, multiple lesions than non-septic shock patients. Regarding clinical outcomes, the septic shock group had longer CRRT duration, vasopressors duration, mechanical ventilator duration and length of ICU stay. (Table 1).

**Fig. 2:**
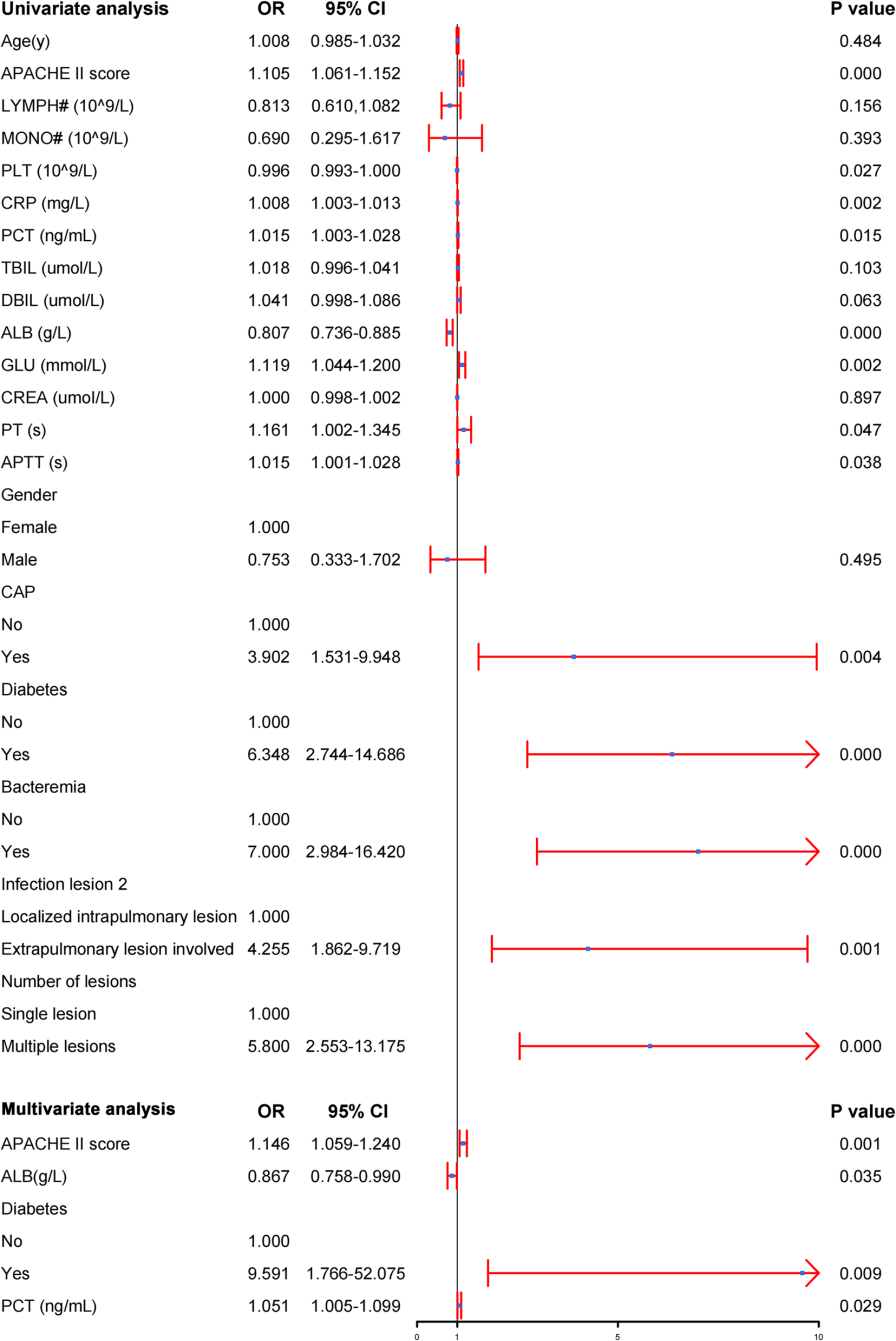
Univariate and multivariate logistic analyses of risk factors associated with septic shock in *hvKP* infections patients. Abbreviations: OR, Odds Ratio; 95% CI, confidence interval; APACHE II score, Acute Physiology and Chronic Health Evaluation II score; LYMPH#, lymphocyte count; MONO#, monocyte count; PLT, platelet; CRP, C-reactive protein; PCT, procalcitonin; TBIL, total bilirubin; DBIL, direct bilirubin; ALB, albumin; GLU, glucose; CREA, creatinine; PT, plasma prothrombin time; APTT, activated partial thromboplastin time; CAP, community acquired pneumonia; *hvKP*, *Hypervirulent Klebsiella pneumoniae*

Univariate analysis showed that APACHE II score, PLT, CRP, PCT, ALB, GLU, PT, APTT, CAP, diabetes, bacteremia, extrapulmonary lesion involved and multiple lesions were risk factors for septic shock. Multivariate logistic analysis showed that increased APACHE II score [odds ratio (OR) = 1.146; 95% confidence interval (CI), 1.059–1.240], decreased ALB level (OR = 0.867; 95%CI, 0.758–0.990), diabetes (OR = 9.591; 95%CI, 1.766–52.075) and high PCT level (OR = 1.051; 95%CI, 1.005–1.099) were independent risk factors for septic shock (Fig. 2).

**Fig. 3:**
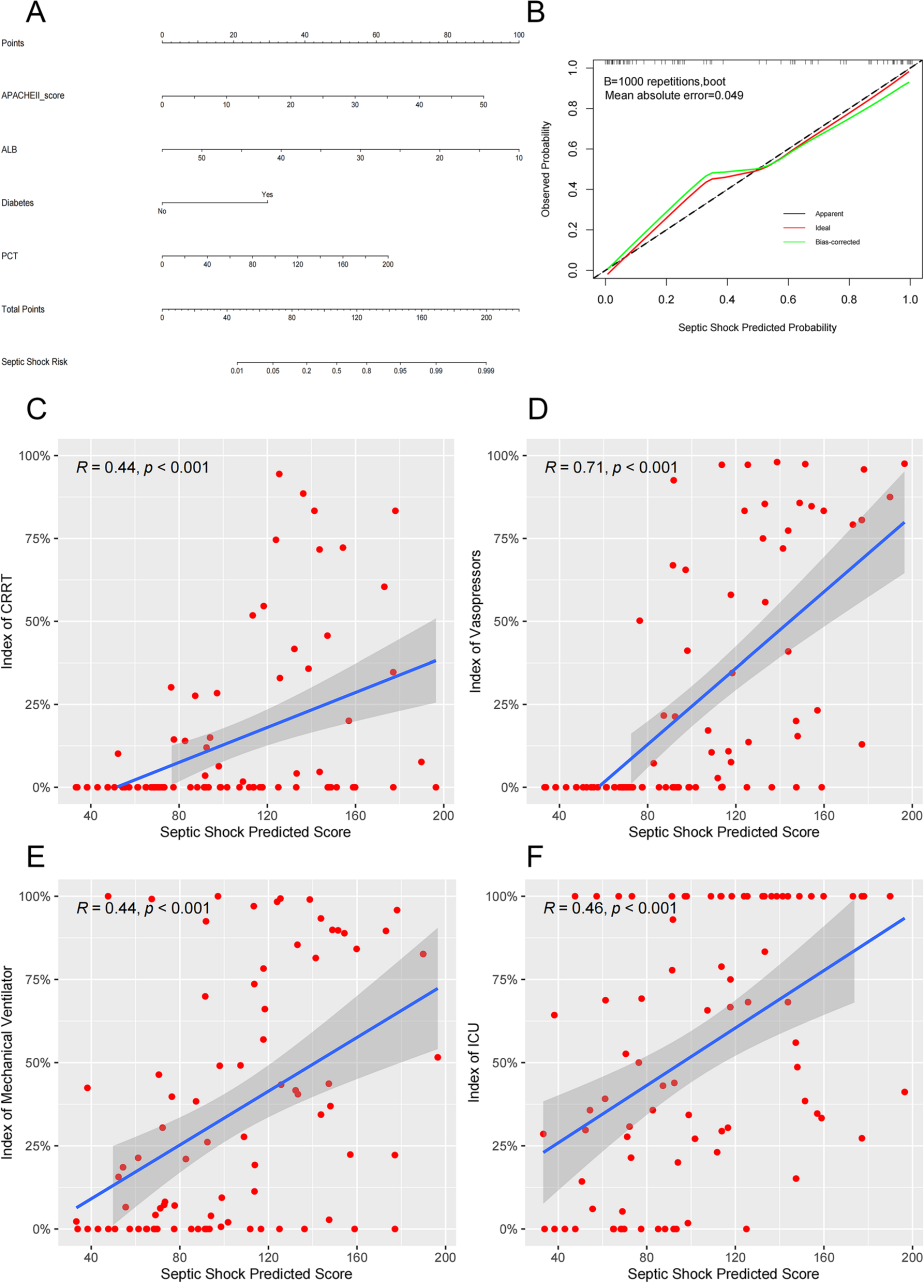
The nomogram, calibration curves and correlation analysis scatter plot of septic shock in *hvKP* infections. **A** Nomogram with the independent risk factors. **B** The calibration curves of the nomogram of septic shock (Mean absolute error = 0.049). **C** Relationships between septic shock predicited score of the nomogram and index of CRRT (CRRT duration/length of hospital stay) (*R* = 0.44, *p* < 0.001). **D** Relationships between septic shock predicited score of the nomogram and index of vasopressors (vasopressors duration/length of hospital stay) (*R* = 0.71, *p* < 0.001). **E** Relationships between septic shock predicited score of the nomogram and index of mechanical ventilator (mechanical ventilator duration/length of hospital stay) (*R* = 0.44, *p* < 0.001). **F** Relationships between septic shock predicited score of the nomogram and index of ICU (length of ICU stay/length of hospital stay) (*R* = 0.46, *p* < 0.001). Abbreviations: APACHE II score, Acute Physiology and Chronic Health Evaluation II score; ALB, albumin; CRRT, continuous renal replacement therapy; ICU, intensive care unit

To assess the probability of septic shock, a nomogram with the independent risk factors was constructed, and the calibration curves of the nomogram showed high consistencies between the predicted and actual septic shock probability (Fig. 3A, B). To further investigate the validation of the nomogram, we calculated the septic shock predicted score and drew a correlation analysis scatter plot with CRRT duration, vasopressors duration, mechanical ventilator duration and length of ICU stay respectively. Positive correlations between predicted scores and indices of CRRT, vasopressor, mechanical ventilator and ICU were observed (Fig. 3C-F).

#### Risk factors associated with ARDS

Seventy-nine (68.1%) patients developed ARDS. The median diagnosis time was 26.00 (18.17, 41.50) hours after admission. No significant difference was found in terms of smoking, alcohol consumption, diabetes, hepatopathy or CKD between the non-ARDS and ARDS groups (*P* > 0.05). Compared with non-ARDS patients, ARDS patients had higher levels of APACHE II score, GLU, PT and APTT, but lower levels of MONO#, ALB. The ARDS patients had significantly higher proportions of CAP, cardiovascular disease, abscesses, hepatic abscesses and extrahepatic lesion involved and multiple pathogens. For clinical outcomes, the ARDS patients had longer CRRT duration, vasopressors duration, mechanical ventilator duration and length of ICU stay. (Table 1).

**Fig. 4:**
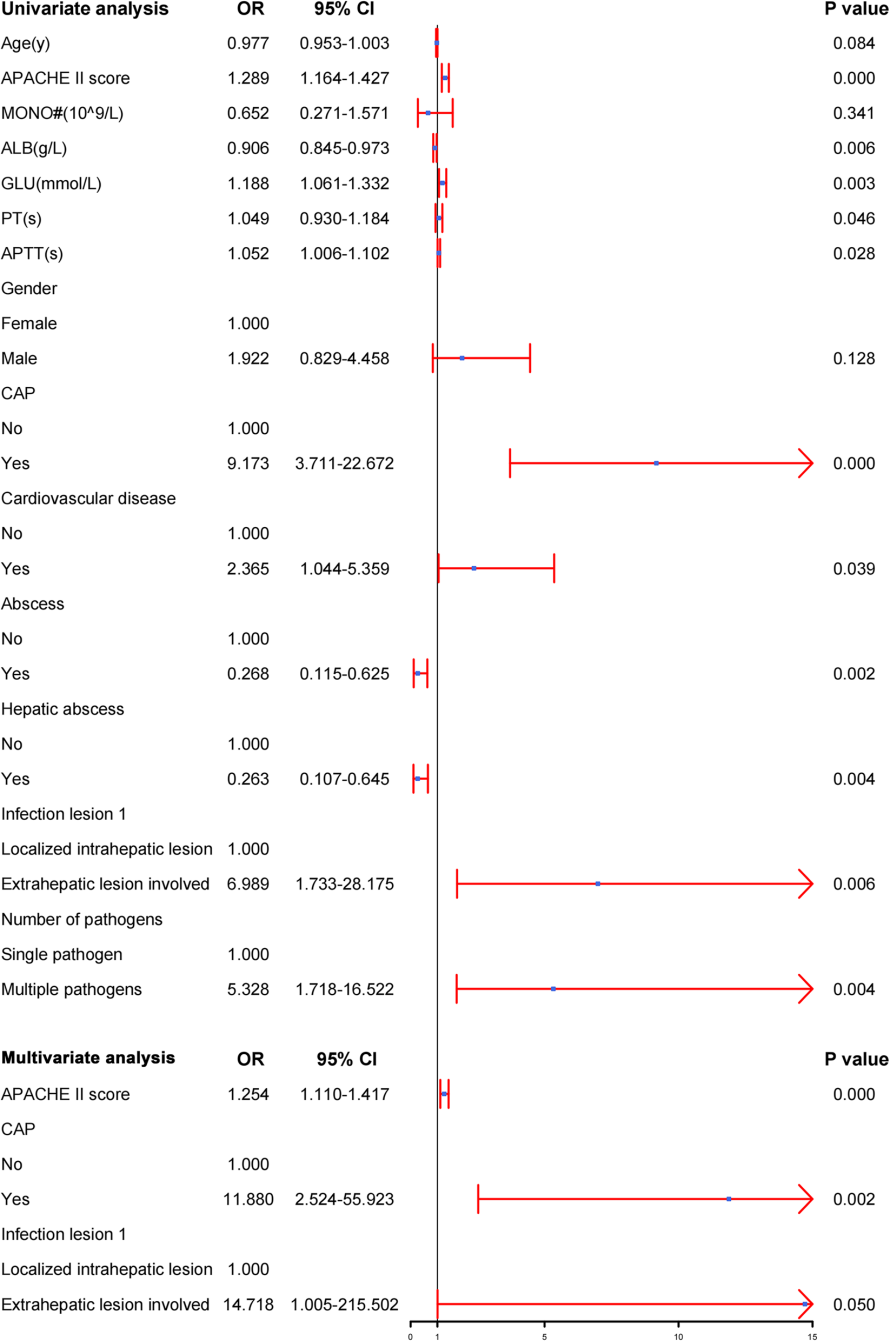
Univariate and multivariate logistic analyses of the risk factors associated with ARDS in *hvKP* infections patients. Abbreviations: OR, Odds Ratio; 95% CI, confidence interval; APACHE II score, Acute Physiology and Chronic Health Evaluation II score; MONO#, monocyte count; ALB, albumin; GLU, glucose; PT, plasma prothrombin time; APTT, activated partial thromboplastin time; CAP, community acquired pneumonia; *hvKP*, *Hypervirulent Klebsiella pneumoniae*

Univariate analysis showed that APACHE II score, ALB, GLU, PT, APTT, CAP, cardiovascular disease, abscess, hepatic abscess, extrahepatic lesion involved and multiple pathogens were significantly associated with ARDS. Multivariate logistic analysis showed that increased APACHE II score (OR = 1.254; 95% CI, 1.110–1.147), community-acquired pneumonia (CAP) (OR = 11.880; 95% CI, 2.524–55.923), and extrahepatic lesion involved (OR = 14.718; 95% CI, 1.005–215.502) were the independent risk factors for ARDS (Fig. 4).

**Fig. 5:**
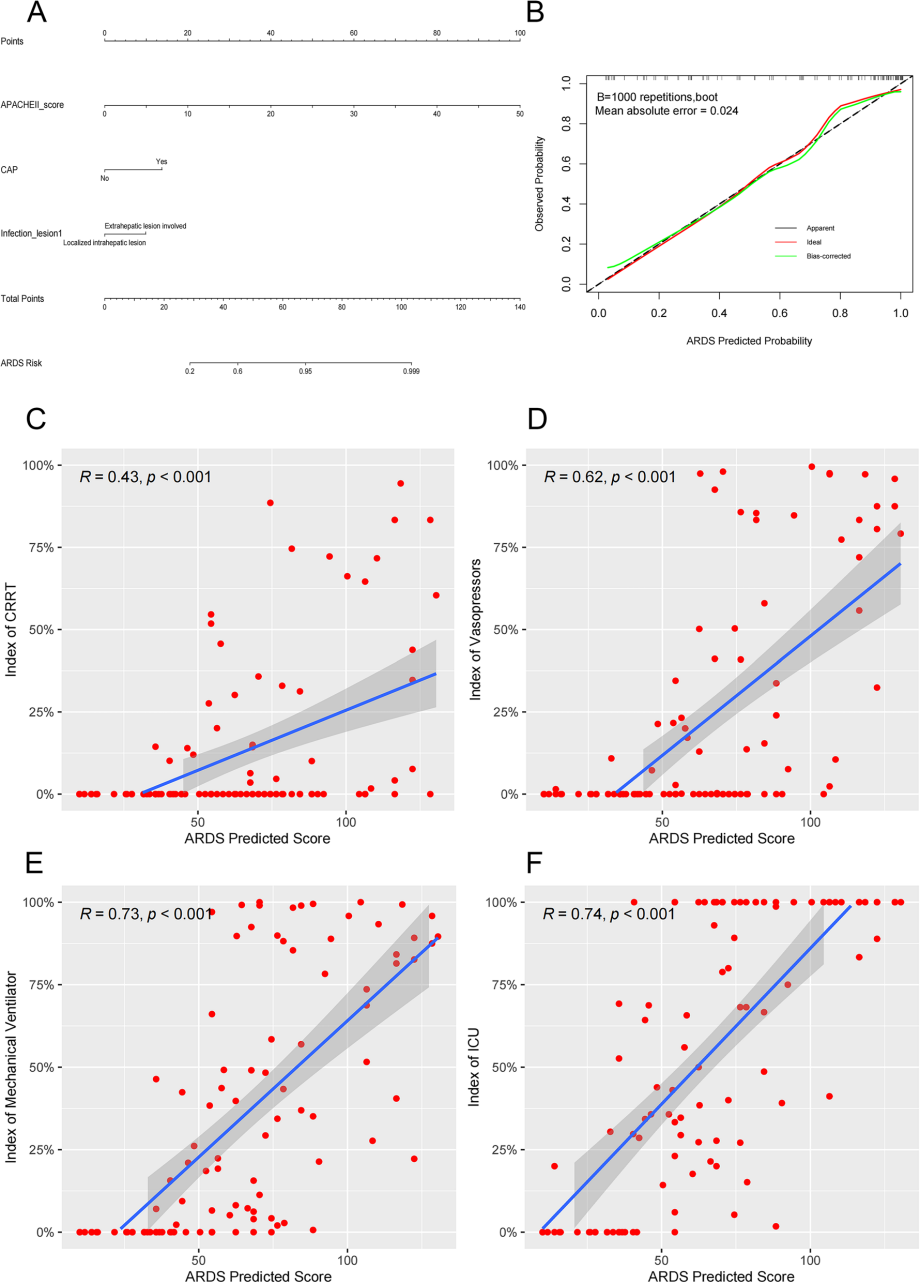
The nomogram, calibration curves and correlation analysis scatter plot of ARDS in *hvKP* infections. **A** Nomogram with the independent risk factors. **B** The calibration curves of the nomogram of ARDS (Mean absolute error = 0.024). **C** Relationships between ARDS predicited score of the nomogram and index of CRRT (CRRT duration/length of hospital stay) (*R* = 0.43 *p* < 0.001). **D** Relationships between ARDS predicited score of the nomogram and index of vasopressors (vasopressors duration/length of hospital stay) (*R* = 0.62, *p* < 0.001). **E** Relationships between ARDS predicited score of the nomogram and index of mechanical ventilator (mechanical ventilator duration / length of hospital stay) (*R* = 0.73, *p* < 0.001). **F** Relationships between ARDS predicited score of the nomogram and index of ICU (length of ICU stay/ length of hospital stay) (*R* = 0.74, *p* < 0.001). Abbreviations: GLU, glucose; CAP, community acquired pneumonia; ARDS, acute respiratory distress syndrome. CRRT, continuous renal replacement therapy, ICU, intensive care unit time

To assess the probability of ARDS, a nomogram with the independent risk factors was constructed, and the calibration curves of the nomogram showed high consistencies between the predicted and actual ARDS probability (Fig. 5A, B). To further validate the nomogram, we calculated the ARDS predicted score and drew a correlation analysis scatter plot with CRRT duration, vasopressors duration, mechanical ventilator duration and length of ICU stay. Positive correlations between predicted scores and indices of CRRT, vasopressors, mechanical ventilator and ICU were observed (Fig. 5C-F).

### Risk factors associated with 30-day mortality

The 30-day all-cause mortality rate in patients with *hvKP* infections was 28.4% (33/116). There were no significant differences in the percentages of diabetes, hepatopathy, CKD and cardiovascular disease between the survivors and non-survivors (*P* > 0.05). Compared with survivors, levels of APACHE II score, CREA, PT and APTT were higher in non-survivors, while the level of ALB was lower. Furthermore, our results revealed that the non-survivors group had significantly higher proportions of smoking, alcohol consumption, CAP, septic shock and ARDS. The non-survivors had longer CRRT duration, vasopressors duration and mechanical ventilator duration, whereas the length of hospital stay was shorter for non-survivors. (Table 2).

**Table 2:** Clinical variables associated with 30-day mortality of *hvKP* infections

Characteristics	Total *n* = 116	Survivors *n* = 83(71.6%)	Non-survivors *n* = 33(28.4%)	*P* value
	**(Mean ± SD) or Median (IQR) or n (%)**			
Age(y)	55.94 ± 15.93	56.65 ± 15.17	54.15 ± 17.82	0.448
Gender				0.527
Female	33(28.4)	25(30.1)	8(24.2)	
Male	83(71.6)	58(69.9)	25(75.8)	
Smoking				0.035
No	89(76.7)	68(81.9)	21(63.6)	
Yes	27(23.3)	15(18.1)	12(36.4)	
Alcohol consumption				0.004
No	96(82.8)	74(89.2)	22(66.7)	
Yes	20(17.2)	9(10.8)	11(33.3)	
CAP				0.008
No	35(30.2)	31(37.3)	4(12.1)	
Yes	81(69.8)	52(62.7)	29(87.9)	
Diabetes				0.315
No	75(64.7)	56(67.5)	19(57.6)	
Yes	41(35.3)	27(32.5)	14(42.4)	
Hepatopathy				0.275
No	79(68.1)	59(71.1)	20(60.6)	
Yes	37(31.9)	24(28.9)	13(39.4)	
CKD				0.856
No	108(93.1)	78(94.0)	32(90.9)	
Yes	8(6.9)	5(6.0)	3(9.1)	
Cardiovascular disease				0.881
No	62(53.4)	44(53.0)	18(54.5)	
Yes	54(46.6)	39(47.0)	15(45.5)	
Septic shock				0.000
No	69(59.5)	61(73.5)	8(24.2)	
Yes	47(40.5)	22(26.5)	25(75.8)	
ARDS				0.000
No	37(31.9)	36(43.4)	1(3.0)	
Yes	79(68.1)	47(56.6)	33(97.0)	
APACHE II score	20.04 ± 12.51	14.37 ± 8.15	34.30 ± 9.95	0.000
WBC (10^9^/L)	14.88(9.58,20.98)	14.93(10.50,17.73)	14.44(6.99,25.98)	0.947
NEUT# (10^9^/L)	12.45(7.87,17.22)	12.75(9.03,15.51)	10.64(4.84,22.65)	0.632
LYMPH# (10^9^/L)	1.03(0.55,1.77)	1.06(0.60,1.77)	1.01(0.44,1.79)	0.515
MONO# (10^9^/L)	0.67(0.29,0.99)	0.70(0.35,1.01)	0.40(0.11,0.93)	0.076
HGB (g/L)	127.50(112.00,141.00)	126.00(113.00,140.00)	128.00(111.50,146.50)	0.446
PLT (10^9^/L)	207.50(122.00,303.25)	204.00(142.00,301.00)	211.00(94.50,308.50)	0.621
CRP(mg/L)	101.62(6.14,184.64)	105.79(5.00,192.48)	96.93(7.62,164.88)	0.960
PCT(ng/mL)	3.33(0.22,30.83)	2.03(0.11,26.48)	14.22(1.065,36.49)	0.064
ALT(U/L)	44.75(19.38,75.08)	37.00(18.63,74.23)	50.00(25.18,78.55)	0.185
AST(U/L)	38.0(23.5,89.75)	36.00(22.85,75.00)	48.00(29.9,152.00)	0.101
TBIL(μmol/L)	13.40(9.48,21.98)	13.10(9.60,19.16)	14.00(8.50,28.25)	0.520
DBIL(μmol/L)	5.89(3.70,10.64)	5.46(3.35,9.90)	7.34(4.75,12.60)	0.109
ALB(g/L)	34.35 ± 6.86	35.67 ± 6.82	31.16 ± 5.94	0.002
GLU(mmol/L)	9.42(6.92,15.90)	9.01(6.95,13.00)	11.76(6.16,18.25)	0.391
CREA(μmoI/L)	86.50(66.45,135.64)	81.90(62.55,113.95)	112.45(83.34,252.23)	0.007
PT(s)	12.40(11.40,14.10)	12.00(10.98,13.73)	13.6(11.65,16.10)	0.006
APTT(s)	30.70(24.30,42.30)	28.85(23.65,35.28)	37.40(28.65,45.95)	0.005
FIB(g/L)	4.13(2.80,6.13)	4.36(2.80,6.26)	3.93(2.57,5.93)	0.620
PO2(mmHg)	100.50(74.38,136.63)	99.40(74.00,138.10)	101.00(78.90,135.90)	0.965
PCO2(mmHg)	33.55(26.98,40.53)	33.80(29.55,38.15)	33.00(24.10,47.70)	0.709
OI	242.00(158.96,324.17)	251.67(160.00,330.30)	218.30(155.17,306.52)	0.279
FiO2(%)	51.02 ± 16.80	48.87 ± 15.73	55.11 ± 18.26	0.112
PEEP(cmH2O)	6.49 ± 2.81	6.43 ± 2.54	6.57 ± 3.28	0.885
LAC(mmol/L)	2.40(1.68,4.88)	2.13(1.61,4.17)	2.73(1.09,5.46)	0.166
Abscess				0.010
No	82(70.7)	53(63.9)	29(87.9)	
Yes	34(29.3)	30(36.1)	4(12.1)	
Hepatic abscess				0.073
No	89(76.7)	60(72.3)	29(87.9)	
Yes	27(23.3)	23(27.7)	4(12.1)	
Pulmonary abscess				1.000
No	113(97.4)	81(97.6)	32(97.0)	
Yes	3(2.6)	2(2.4)	1(3.0)	
Bacteremia				0.117
No	76(65.5)	58(69.9)	18(54.5)	
Yes	40(34.5)	25(30.1)	15(44.5)	
Infection lesion 1				0.252
Localized intrahepatic lesion	11(9.5)	10(12.0)	1(3.0)	
Extrahepatic lesion involved	105(90.5)	73(88.0)	32(97.0)	
Infection lesion 2				0.926
Localized intrapulmonary lesion	50(43.1)	36(43.4)	14(42.4)	
Extrapulmonary lesion involved	66(56.9)	47(56.6)	19(57.6)	
Number of lesions				0.057
Single lesion	72(62.1)	56(67.5)	16(48.5)	
Multiple lesions	44(37.9)	27(32.5)	17(51.5)	
Number of pathogens				0.172
Single pathogen	81(69.8)	61(73.5)	20(60.6)	
Multiple pathogens	35(30.2)	22(26.5)	13(39.4)	
Initial antimicrobial regimens				0.009
Penicillin/third-generation cephalosporins	12(10.5)	11(13.6)	1(3.0)	
(Penicillin/third-generation cephalosporins) + beta-lactamase inhibitor	49(43.0)	33(40.7)	16(48.5)	
Carbapenems	24(21.1)	15(18.5)	9(27.3)	
Quinolones/second-generation cephalosporins	13(11.4)	11(13.6)	2(6.1)	
(Penicillin/third-generation cephalosporins) + (quinolones/aminodycosides)	4(3.5)	2(2.5)	2(6.1)	
(Penicillin/third-generation cephalosporins) + beta-lactamase inhibitor + (quinolones/aminodycosides)	9(7.9)	9(11.1)	0(0.0)	
Carbapenems + quinolones	3(2.6)	0(0.0)	3(9.1)	
Number of antimicrobials				0.632
Single antimicrobial	101(88.6)	73(90.1)	28(84.8)	
Combined antimicrobials	13(11.4)	8(9.9)	5(15.2)	
CRRT duration(d)	0.00(0.00,1.28)	0.00(0.00,0.00)	0.75(0.00,3.56)	0.001
Vasopressors duration(d)	0.00(0.00,4.24)	0.00(0.00,1.08)	3.87(1.39,8.40)	0.000
Mechanical ventilator duration(d)	2.07(0.00,8.79)	0.75(0.00,8.79)	4.67(1.77,8.86)	0.005
Length of ICU stay(d)	6.50(0.25,15.00)	7.00(0.00,17.00)	5.00(2.50,10.50)	0.926
Length of hospital stay(d)	18.00(9.00,33.00)	24.00(15.00,37.00)	6.00(3.00,12.00)	0.000

**Fig. 6:**
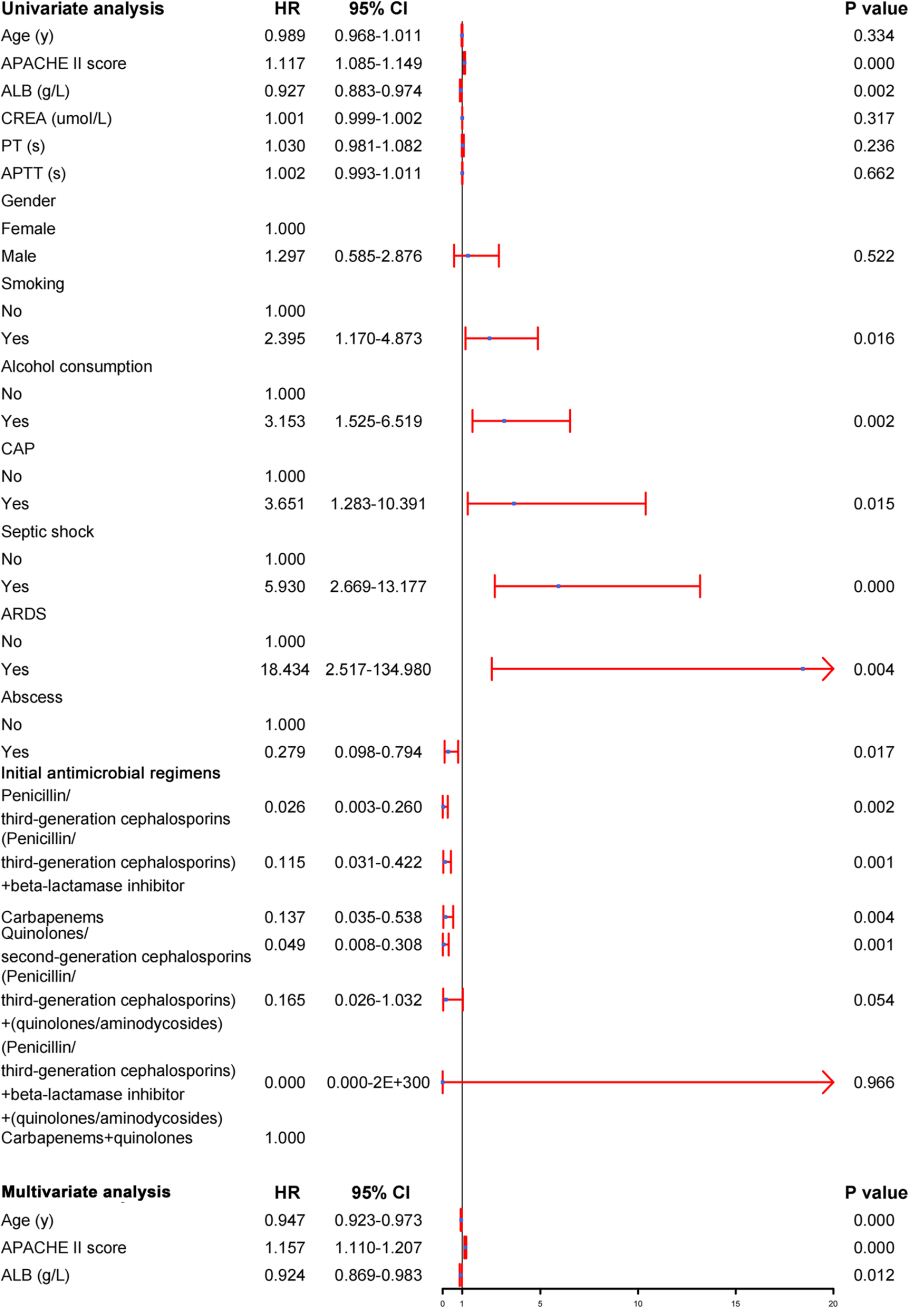
Univariate and multivariate cox analyses on variables for the prediction of 30-day all-cause mortality of *hvKP* infection patients. Abbreviations: HR, Hazard ratio; 95% CI, confidence interval; APACHE II score, Acute Physiology and Chronic Health Evaluation II score; ALB, albumin; CREA, creatinine; PT, plasma prothrombin time; APTT, activated partial thromboplastin time; CAP, community acquired pneumonia; ARDS, acute respiratory distress syndrome

As shown in Fig. 6, APACHE II score, ALB, smoking, alcohol consumption, CAP, septic shock, ARDS, abscess and initial antimicrobial regimens were significantly associated with 30-day mortality. According to multivariate analysis results, younger age [hazard ratio (HR) = 0.947; 95% CI, 0.923–0.973)], increased APACHE II score (HR = 1.157; 95% CI, 1.110–1.207), and lower ALB (HR = 0.924; 95% CI, 0.869–0.983) were independent risk factors for 30-day mortality.

**Fig. 7:**
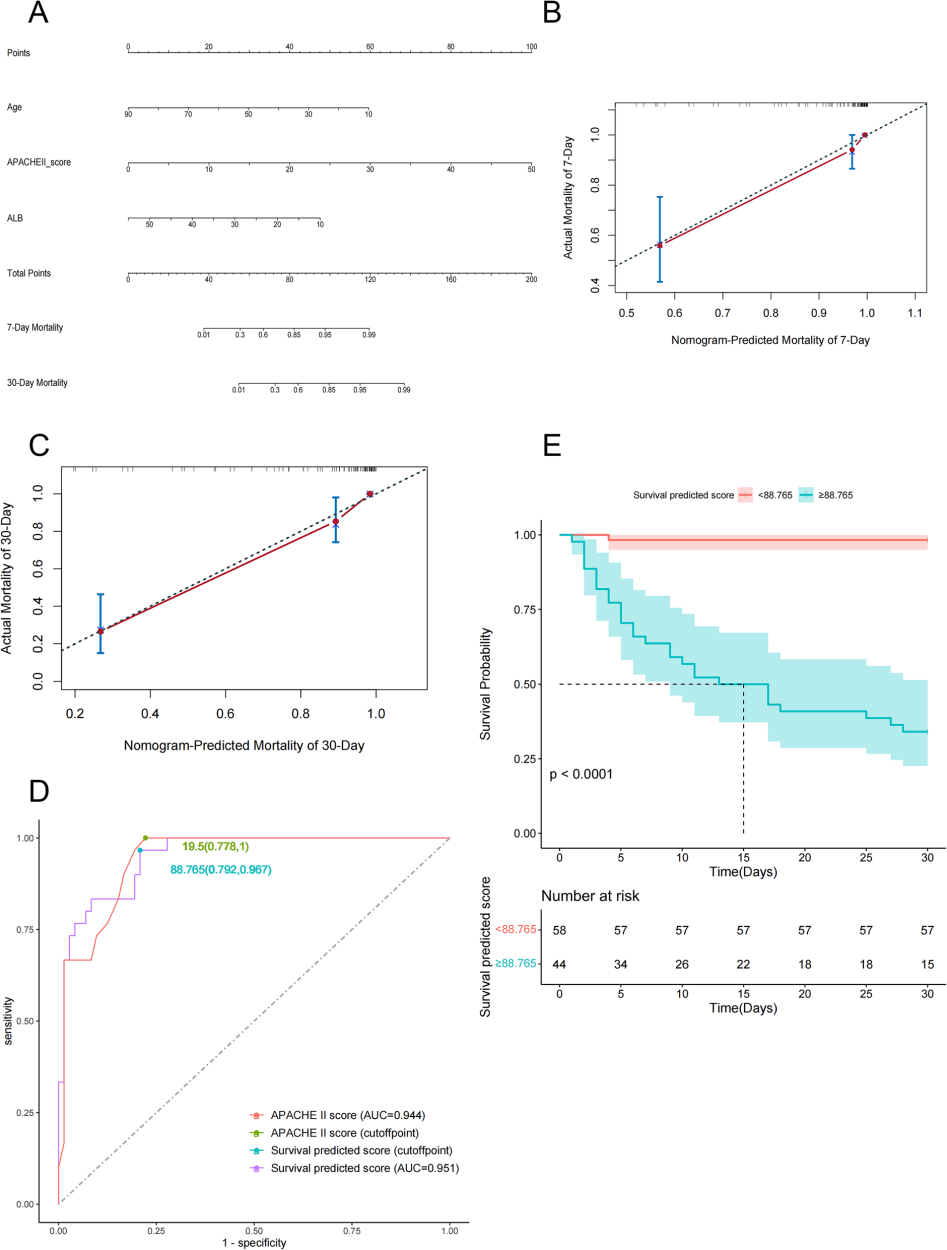
The nomogram, calibration curves of assessment models of the 7-day and 30-day all-cause mortality, ROC curve and K-M curve of assessment models of the 30-day all-cause mortality in *hvKP* infections. **A** Nomograms of 7-day and 30-day mortality with the independent risk factors. **B** Calibration curve of the nomogram of 7-day mortality. **C** Calibration curve of the nomogram of 30-day mortality. The light blue line indicates the ideal reference line where predicted mortality would match the actual mortality. The red dots are calculated by bootstrapping (resample: 1000) and represent the performance of the nomogram. The closer the solid red line is to the light blue line, the more accurately the model predicts mortality. **D** ROC curves of survival predicted score and APACHE II score. survival predicted score: cutoff value = 88.765 (AUC = 0.951 specificity = 0.792, sensitivity = 0.967); APACHE II score: cutoff value = 19.5 (AUC = 0.944, sensitivity = 0.778, specificity = 1.000). **E** K-M curves of survival predicted score. The survival rate of high-risk group (survival predicted score ≥ 88.765) was significantly lower than that of low-risk group (survival predicted score < 88.765) (34.1% VS 98.3%, *p* < 0.0001). Abbreviations: APACHE II score, Acute Physiology and Chronic Health Evaluation II score; (ROC) curve, receiver operating characteristic curve; ARDS, acute respiratory distress syndrome. AUC, area under (the) curve; K-M curve, Kaplan–Meier curve

We constructed a nomogram of 7-day and 30-day mortality and the calibration curves showed high consistencies between the predicted and actual mortality (Fig. 7A-C). Furthermore, to compare the predictive effects of the prognostic model of 30-day mortality and the APACHE II score, we drew ROC curves of the survival predicted score and APACHE II score. The results showed that the cut-off value of the survival predicted score was 88.765 [area under the curve (AUC) 0.951, specificity 0.792, sensitivity 0.967], and the cut-off value of the APACHE II score was 19.5 (AUC 0.944, sensitivity 0.778, specificity 1.000) (Fig. 7D). We divided the survival predicted score into low-risk group (survival predicted score < 88.765) and high-risk group (survival predicted score ≥ 88.765), and then drew a K-M curve, which showed that the survival rate of the high-risk group was significantly lower than that of the low-risk group (34.1% vs. 98.3%, *p* < 0.0001) (Fig. 7E).

## Discussions

*hvKP* infections have emerged as a major clinical and public health threat over the past decade [1, 10, 18, 19]. As clinical manifestations of *hvKP* infections are nonspecific, and differentiation of *hvKP* strains is mainly based on phenotypic and genotypic features without universal standards, it is difficult to identify *hvKP* infections early. Currently, knowledge of risk factors for disease severity and mortality remains limited. Few studies have investigated the risk factors for mortality in patients with *hvKP* infections, while no study has explored the risk factors or a prognostic model for disease severity. Since genetic testing is not easy to perform clinically, we summarize the routine clinical parameters to investigate risk factors associated with disease severity and 30-day mortality and construct the prognostic models. This may be the first study to report the risk factors for the severity of *hvKP* infection, and prognostic models of disease severity and 30-day mortality clinically.

Some *hvKP-*infected patients develop septic shock, ARDS. In our study, the median diagnosis times of septic shock and ARDS were 11.63 (4.00, 26.00) and 26.00 (18.17, 41.50) hours after admission, respectively. Furthermore, patients with septic shock and ARDS had longer CRRT duration, vasopressor duration, mechanical ventilator duration and length of ICU stay. The results suggest that septic shock and ARDS are reasonable predictors for assesssing disease severity in patients with *hvKP* infections.

Multivariate logistic analysis showed that increased APACHE II score, lower ALB, diabetes, high PCT were independent risk factors for septic shock. Studies on the correlation between APACHE II score and severity of *hvKP* infections have not been found. A sepsis patient’s serum ALB can decrease due to various factors including hypermetabolic state, gastrointestinal dysfunction, capillary leakage [20]. There is a causal relationship between hypoalbuminemia and an increased risk of primary and secondary infections, hypoalbuminemia has an effect on the pharmacokinetics and pharmacodynamics of anti-infective drugs, which in turn affects the clinical outcome of infections [21]. Hematocrit (HCT)-ALB difference can be a potential predictor for the prognosis of elderly sepsis patients [20]. In addition, lower ALB is a risk factor for elderly people with bacterial infections [22], and early infusion of albumin seems to reduce the mortality of patients with sepsis [23, 24]. ALB replacement in addition to crystalloids improves the haemodynamics of patients with severe sepsis during the first 7 days [25]. Ongoing research on the ALB administration supports the potential for ALB to improve sepsis survival [23]. Therefore, it is suggested that correcting hypoalbuminemia possibly reduces the risk of *hvKP* infections progressing to septic shock, and improves the clinical outcome of *hvKP* infections. Diabetes mellitus is considered a significant risk factor for acquiring *hvKP* infections [26–29], which primarily affects male individuals aged 55–60 years [30]. Diabetes is an independent risk factor for *KP* pyogenic liver abscess [31], as poor glycaemic control might impair neutrophil phagocytosis and promote pathogen growth in tissues, while metabolic disturbances might negatively affect the liver [32, 33]. Moreover, diabetes, which is more likely to progress *hvKP* infections, especially *hvKP*-bloodstream infections (BSIs) [26, 28], is an independent risk factor for *hvKP*-BSIs [12]. No studies have been found on the association of PCT with the severity of *hvKP* infections. However, the PCT level has been shown to be significantly higher in *hvKP* group compared with *cKP* group [34]. And PCT has been a prognostic biomarker in patients with severe sepsis and septic shock [35]. In addition, serum procalcitonin ≥ 5 ng/mL was found to be associated with 30-day mortality of carbapenem-resistant *KP* infections [36]. Our research shows that PCT [19.07(2.95,42.01) ng/mL] is an independent risk factor for septic shock, indicating that PCT is one of the factors predicting the risk of septic shock in patients with *hvKP* infections.

Multivariate analysis showed that increased APACHE II score, CAP and extrahepatic lesion involved were independent risk factors for ARDS. The APACHE II score is also an independent factor predicting septic shock, so the APACHE II score is very important for the evaluation of *hvKP* infections. Furthermore, *hvKP* infections are usually community-acquired [3, 37, 38], CAP has been showed to be associated with high mortality in patients with *hvKP* infections [39]. Patients with *KP* pyogenic liver abscesses with sepsis have higher rates of septic shock and acute respiratory distress syndrome [40]. Severe *hvKP* infections with pyogenic liver abscesses in healthy adults have been reported previously [10, 31, 37, 41]. Moreover, liver abscess is a significant risk factor for *hvKP* infecitons [42], and abscess has been identified as an independent predictor for associated with *hvKP*-BSIs [43]. Nevertheless, our study revealed that *hvKP* infections with extrahepatic lesion involved were more serious (OR = 14.718), which seems to be inconsistent with previous results. Usually, due to the good permeability of the hepatic sinusoid of the liver, it can promote material exchange between liver cells and blood flow, which is more likely to cause bacteraemia and accelerate the spread of lesions. When the foci of *hvKP* infections is limited to the liver, which may be related to the immune function of the liver. As a line of defence for immunity, the liver causes a localized lesion and reduces the transfer and dissemination of bacteria to a certain extent, thus reducing the occurrence of bacteraemia and the progression of ARDS.

We constructed prognostic models to assess disease severity, validated the effects of these models, and performed correlation analyses between model scores and clinical outcomes including CRRT duration, vasopressors duration, mechanical ventilator duration and length of ICU stay. Since there were not enough additional cases, only internal validation was performed in this study, and the matching degree of internal validation was good. In the correlation analysis between scores of *hvKP* infections severity (septic shock, ARDS) and CRRT duration, vasopressors duration, mechanical ventilator duration, and length of ICU stay, the correlation coefficient R (0.43–074) indicated that the correlation was moderately positive. Therefore, septic shock and ARDS are suitable as observation indicators for evaluating the condition of *hvKP* infections. Due to the small sample size of cases included in this study, further clinical research is needed for verification.

The 30-day all-cause mortality of *hvKP*-infected patients in our study was 28.4%, which is close to previously reported data (4.5%-37.1%) [12, 44–47]. In our study, younger age, increased APACHE II score, and decreased ALB level were independent risk factors for 30-day mortality. It has been noted that the detection rate of *hvKP* among the *KP* isolates increases in the elderly individuals, indicating that ageing can be an elevated risk for *hvKP* infections [26, 28, 48, 49], but age is not statistically significant for hypermucoviscous *KP* infections [27]. The median age of nonsurvivors in our study was 54.15 ± 17.82 years, and younger age was a risk factor for increased mortality, which may be contributed by the violent inflammatory reaction in young people, most of whom showed multiple organ dysfunction, septic shock and ARDS. This point also reminds clinicians that in the face of *hvKP* infections in young and middle-aged adults, modulating the host immune response may be an effective regimen to reduce mortality. In our study, the APACHE II score (HR = 1.157) in the nonsurvivors group was 34.30 ± 9.95. Previous studies have shown that a higher APACHE II score is correlated with a higher 30-day all-cause mortality rate of *hvKP* infections [12, 50], which is consistent with our findings. A low ALB level predicts worse outcomes for patients with BSIs caused by Enterobacteriaceae [51], and for mortality in liver transplant recipients with gram-negative bacilli (GNB) bacteraemia [52].These results are consistent with our findings.

Although most *hvKP* strains are rarely resistant to common antimicrobials, antibiotic-resistant *hvKP* isolates have been increasing over the past few years, and no literature has yet reported which antimicrobial is the most effective [4, 53–55]. In our study, the initial antimicrobial regimens included piperacillin/third-generation of cephalosporins (10.5%), piperacillin/third-generation of cephalosporins combined with beta-lactamase inhibitor (43.0%), carbapenems (21.1%), quinolones/second-generation of cephalosporins (11.4%), piperacillin/third-generation cephalosporins combined with quinolones/aminodycosides (3.5%), piperacillin/third-generation cephalosporins combined with beta-lactamase inhibitor and quinolones/aminodycosides (7.9%), and carbapenems combined with quinolones (2.6%). Multiple comparisons have revealed that carbapenems combined with quinolones had higher mortality rates than piperacillin/third-generation cephalosporins combined with beta-lactamase inhibitor and quinolones/aminodycosides (100.0% vs. 0.0%, *P* = 0.005) and piperacillin/third-generation cephalosporins (100.0% vs. 8.3%, *P* = 0.009). Univariate analysis suggested that, compared with the prognosis of the combination of carbapenems and quinolones (HR = 1.000), piperacillin/third-generation cephalosporins (HR = 0.026; 95%CI, 0.003–0.260; *P* = 0.002), piperacillin/third-generation cephalosporins combined with beta-lactamase inhibitor (HR = 0.115; 95%CI, 0.031–0.422; *P* = 0.001), carbapenems (HR = 0.137; 95%CI, 0.035–0.538; *P* = 0.004) and quinolones/second-generation cephalosporins (HR = 0.049; 95%CI, 0.008–0. 308; *P* = 0.001) conferred better prognosis. Thus, carbapenems seem not to be the first choice for *hvKP* infections unless they are chosen based on drug sensitivity tests. However, due to the insufficient number of cases, further verifications in prospective studies are needed.

We constructed the prognostic models of 30-day mortality with the variables including age, APACHE II score and ALB level. According to ROC curves of the survival predicted score and APACHE II score, we took survival predicted score = 88.765 as the cut-point, and drew the K‒M curves. K‒M survival analysis showed that the 30-day mortality of the high-risk group (score ≥ 88.765) was significantly higher than that of the low-risk group (score < 88.765) (34.1% vs. 98.3%, *p* < 0.0001). The model not only had a good internal validation effect, but also was consistent with previous results.

There were several limitations in our research. Firstly, it was a retrospective study and the sample was quite small. In addition, it was a regional study that all the cases came from Dongguan, which was a labor-intensive city with a large inflow of young people. Finally, external validations of the prognostic models were not feasible due to a lack of additional data. Further investigations are required to confirm these results.

## Conclusions

In this retrospective study, increased APACHE II score, decreased ALB, diabetes, higher PCT, CAP and extrahepatic lesion involved were identified as independent risk factors for septic shock and ARDS in patients with *hvKP* infections. The prognostic models constructed for disease severity with these conventional parameters, were significantly correlated with clinical outcomes, making them potentially practical for clinicians. Moreover, younger age, increased APACHE II score, and lower ALB were independent risk factors for 30-day all-cause mortality. The prediction model for 30-day mortality had a good validation effect. In summary, we constructed the prognostic models for disease severity and 30-day mortality in patients with *hvKP* infections, and the models were helpful for making more practical and effective therapeutic decisions.

## Data Availability

Please contact Yi Chen if someone wants to request the data from this study. Authors have not specified any datasets in this data configuration file. The data that have been used is confidential. It is collected by the authors in the hospital medical record system, and have not been published anywhere. Due to the sensitive nature of the questions asked in this study, survey respondents were assured raw data would remain confidential and would not be shared.

## Abbreviations

*hvKP*: *Hypervirulent Klebsiella pneumoniae*
*cKP*: *C* *lassic Klebsiella pneumoniae*
ARDS: Acute respiratory distress syndrome
APACHE II: Acute Physiology and Chronic Health Evaluation II
CAP: Community-acquired pneumonia
CKD: Chronic kidney disease
WBC: White blood cell count
NEUT#: Neutrophil count
LYMPH#: Lymphocyte count
MONO#: Monocyte count
HGB: Hemoglobin
PLT: Platelet
CRP: C-reactive protein
PCT: Procalcitonin
ALT: Alanine aminotransferase
AST: Aspartate aminotransferase
TBIL: Total bilirubin
DBIL: Direct bilirubin
ALB: Albumin
GLU: Glucose
CREA: Creatinine
PT: Plasma prothrombin time
APTT: Activated partial thromboplastin time
FIB: Fibrinogen
PO2: Partial pressure of oxygen
PCO2: Partial pressure of carbon dioxide
FIO2: Fraction of inspired oxygen
OI: Oxygenation index
PEEP: Positive end expiratory pressure
LAC: Lactate
CRRT: Continuous renal replacement therapy
ICU: Intensive care unit
SD: Standard deviation
IQR: Interquartile range
ROC: Receiver operating characteristic
K-M curve: Kaplan–Meier curve
AUC: Area under (the) curve
OR: Odds ratio
CI: Confidence interval
HR: Hazard ratio
BSIs: Bloodstream infections
GNB: Gram-negative bacilli
